# Neuromuscular function during knee extension exercise after cold water immersion

**DOI:** 10.1186/s40101-017-0144-8

**Published:** 2017-06-23

**Authors:** Hitoshi Wakabayashi, Titis Wijayanto, Yutaka Tochihara

**Affiliations:** 1 Laboratory of Environmental Ergonomics, Faculty of Engineering, Hokkaido University, N13 W8, Kita-ku, Sapporo, 060-8628 Hokkaido Japan; 2 Department of Mechanical and Industrial Engineering, Gadjah Mada University, Yogyakarta, Indonesia; 3 Faculty of Design, Kyushu University, Fukuoka, Japan

**Keywords:** Cold water immersion, Hypothermic skeletal muscle, Electromyography, Mean power frequency

## Abstract

**Background:**

Human adaptability to cold environment has been focused on in the physiological anthropology and related research area. Concerning the human acclimatization process in the natural climate, it is necessary to conduct a research assessing comprehensive effect of cold environment and physical activities in cold. This study investigated the effect of cold water immersion on the exercise performance and neuromuscular function during maximal and submaximal isometric knee extension.

**Methods:**

Nine healthy males participated in this study. They performed maximal and submaximal (20, 40, and 60% maximal load) isometric knee extension pre- and post-immersion in 23, 26, and 34 °C water. The muscle activity of the rectus femoris (RF) and vastus lateralis (VL) was measured using surface electromyography (EMG). The percentages of the maximum voluntary contraction (%MVC) and mean power frequency (MPF) of EMG data were analyzed.

**Results:**

The post-immersion maximal force was significantly lower in 23 °C than in 26 and 34 °C conditions (*P* < 0.05). The post-immersion %MVC of RF was significantly higher than pre-immersion during 60% maximal exercise in 23 and 26 °C conditions (*P* < 0.05). In the VL, the post-immersion %MVC was significantly higher than pre-immersion in 23 and 26 °C conditions during 20% maximal exercise and in 26 °C at 40 and 60% maximal intensities (*P* < 0.05). The post-immersion %MVC of VL was significantly higher in 26 °C than in 34 °C at 20 and 60% maximal load (*P* < 0.05). The post-immersion MPF of RF during 20% maximal intensity was significantly lower in 23 °C than in 26 and 34 °C conditions (*P* < 0.05), and significantly different between three water temperature conditions at 40 and 60% maximal intensities (*P* < 0.05). The post-immersion MPF of VL during three submaximal trials were significantly lower in 23 and 26 °C than in 34 °C conditions (*P* < 0.05).

**Conclusions:**

The lower shift of EMG frequency would be connected with the decrease in the nerve and muscle fibers conduction velocity. To compensate for the impairment of each muscle fibers function, more muscle fibers might be recruited to maintain the working load. This might result in the greater amplitude of EMG after the cold immersion.

## Background

Human adaptability to cold environment has been studied well and summarized in numbers of paper [1–3]. There have been several approaches of the cold adaptation studies using “acclimatization” to the natural climate or “acclimation” to an experimentally induced cold environment, defined in the glossary of the International Union of Physiological Sciences [4]. The seasonal difference [5, 6], populations living in different climate [7–9], or people who work in cold work place [10, 11] have been studied for evaluating the cold acclimatization to the natural cold climate. On the other hand, cold acclimation studies have tested the change in physiological responses following a repeated cold exposure in a laboratory, mostly in resting condition [12, 13]. However, since human would have never acclimatized to cold alone without any physical activity, it is necessary to conduct a research assessing comprehensive effect of the physical activities in cold environment. Additionally, adaptation in the physical performance in cold would be one of the interesting research topics in the research area of physiological anthropology [14].

Concerning the importance of studying the cross adaptation of exercise and cold environment, this manuscript focuses on the performance and physiological characteristics during exercise in cold. There have been several review articles summarizing the human performance and physiological responses in cold environment [14–17]. The electromyography (EMG) technique has been often used for assessing the neuromuscular function during muscle activity in cold. When the skeletal muscle temperature is decreased, a lower shift in the EMG frequency has been uniformly reported [18–22]. However, inconsistent results (increased or decreased) have been observed in the amplitude of EMG [18, 19, 22–24]. The discrepancy might be explained by different experimental protocols including exercise type, cooling procedure, and measured muscle groups.

This study aimed to investigate the effect of cold water immersion on the exercise performance and neuromuscular function of the femoral muscles during maximal and submaximal isometric knee extension exercises.

## Methods

### Participants

Nine healthy males (21.7 ± 0.4 years old; 167.8 ± 2.3 cm height; 61.1 ± 1.8 kg body weights; mean ± SE) participated in this study. All experimental protocols in this study were designed according to the principle of the Helsinki Declaration and approved by the Institutional Review Board of Kyushu University. All participants were informed of the experimental procedures and gave their written informed consent before participation.

### Experimental procedure

**Fig. 1:**
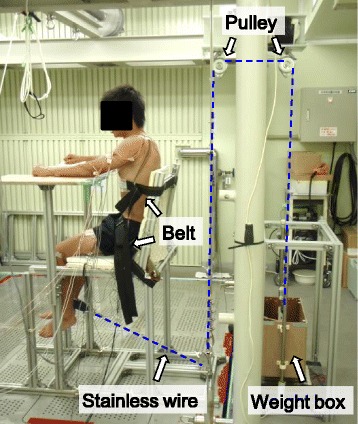
Scheme of the knee extension device

Participants conducted three water temperature conditions (23, 26, and 34 °C) of experiment on separate days in random order. We set 23, 26, and 34 °C water temperature as severe cold, mild cold, and thermoneutral condition, respectively. The 23 °C condition was cold enough to induce shivering, but in 26 °C, no significant shivering was observed as shown in the previous report [12]. At least 1 week before the first test, participants were accustomed to perform one leg knee extension exercise using a specially designed device (Fig. 1). They sat on an aluminum-framed chair with cushion sheet, and their waist and chest were tied to the seat with polyester fiber belts. Their right ankle were attached to an end of stainless wire with a fiber belt, and the another end of wire was attached to a load cell (LUB-200 KB; Kyowa Electronic Instruments Co., Ltd., Japan) or weights thorough some pulley blocks.

Participants came to the laboratory at least 1 h before starting experiment and rested in a thermostatic chamber controlled at 27 °C and 50% relative humidity wearing ordinary swimming trunks. Before immersion, they performed maximal voluntary contraction (MVC) of isometric one leg extension for 5 s with 100° knee joint angles. To motivate them into achieving maximal contraction levels, verbal encouragement by the instructors was provided. The force signal measured by the load cell were transferred to digital data using an A/D converter (SBL21-512, Medical Try System, Japan) and were recorded using a personal computer (Think Centre A51 8425-51J. IBM, Japan) with a 1-kHz sampling rate. Then, they performed 30-s submaximal isometric knee extension at three different exercise intensities (20, 40, and 60% maximal load). They were asked to extend their knee to lift the weight attached to the end of the stainless wire and keep the position of 100° knee joint angle as stable as possible for 30 s (Fig. 1).

Following the pre-immersion leg extension trials, participants were lowered into the water to their chest level using an electric winch attached to the movable floor, and they remained at rest on the chair in a thermostatic water tank for 60 min. After the immersion, participants were pulled out from the water, and they performed post-immersion leg extension trials (maximal and submaximal), in the same protocol as the pre-immersion trials described above.

### Measurements and analysis

**Fig. 2:**
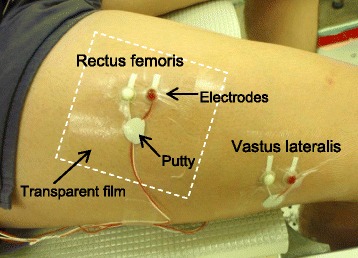
Setting of surface EMG electrodes and waterproof technique using putty and transparent film

The muscle activity of the rectus femoris (RF) and vastus lateralis (VL) were measured during the experiment using surface electromyography (EMG). A pair of 5 mm-diameter silver-silver-chloride surface EMG electrodes (Ag/AgCl Skin Electrode, NT-611T; Nihon Kohden Corp., Japan) was placed on the middle of each muscle belly. A reference electrode was placed on the clavicle. The skin cuticle was removed and cleaned with alcohol wipes so that the inter-electrode impedance was less than 20 kΩ. The electrodes were covered with transparent waterproof film (Dressing tape MA-E100-A; Kyowa Limited., Japan) and putty for blocking water leakage through the lead wire of the electrodes (Fig. 2).

The raw EMG signals during the maximal and each submaximal knee extension trials were amplified with a multi-channel bioamplifier (Biotop 6R12-4; NEC San-ei Co. Ltd., Japan) and were filtered using band-pass filters ranging from 20 to 500 Hz. The filtered EMG signals were transferred to digital data using an A/D converter (SBL21-512, Medical Try System., Japan) and were recorded using a personal computer (Think Centre A51 8425-51J. IBM, Japan) with a 1-kHz sampling rate. The root mean square (RMS) was calculated every 512 points of EMG data. The peak 1024 msec RMS values of each muscle MVC were selected as normalizing values (100%). The muscle activity level for each submaximal knee extension trial was presented as percentages of maximum voluntary contraction (%MVC) every second for each muscle. The power spectrum of EMG signal during the submaximal isometric contraction was evaluated every second using the moving fast Fourier transfer analysis with Hamming window of 1024 points data. Then, mean power frequency (MPF) was calculated to ascertain changes in the frequency component. The %MVC and MPF during the submaximal knee extension were averaged in the range of 5–15 s after starting contraction when the value were stable. The 5–15 s averaged %MVC and MPF were used for the following statistical analysis.

### Statistics

Data (maximal force, %MVC, and MPF) were analyzed by a repeated measures two-way (water temperature × pre-post immersion) analysis of variance (ANOVA). This analysis was conducted separately for each RF and VL muscle and for exercise intensities. After determining the main effects, pair-wise post hoc tests were conducted between pre- and post-immersion at each water temperature (Fisher’s post hoc least significant difference test) and among water temperature conditions within pre- and post-immersion (paired Student’s *t* tests). Significant differences were established at *P* < 0.05. All data were presented as mean values and standard error (SE).

## Results

### Maximal knee extension

Maximal force during the maximal voluntary knee extension pre- and post-immersion, and post-immersion %MVC in each muscle relative to the pre-immersion MVC are shown in Table 1. The post-immersion maximal force was significantly lower in 23 °C condition than in 26 and 34 °C conditions (*P* < 0.05), while no statistical difference was observed between conditions at pre-immersion trial. Maximal force tended to be lower in post compared to pre-immersion in 23 °C condition (*P* = 0.08), whereas no statistical difference was observed in 26 and 34 °C conditions. The post-immersion %MVC of the RF was significantly greater in 26 °C condition compared to 23 and 34 °C conditions (*P* < 0.05). No statistical difference between conditions was observed in %MVC of the VL.

**Table 1:** Pre- and post-immersion maximal force and post-immersion amplitude of electromyography in each muscle relative to the pre-immersion maximal voluntary contraction

Water temperature condition (°C)	23	26	34
Pre-immersion maximal force (kg)	53.0 (2.7)	53.8 (2.5)	53.9 (2.7)
Post-immersion maximal force (kg)	48.8 (2.9)^b, c^	52.7 (2.9)^a^	52.7 (3.4)^a^
Post-immersion %MVC in the RF (%)	98.8 (5.6)^b^	116.6 (4.6)^a, c^	94.3 (7.5)^b^
Post-immersion %MVC in the VL (%)	102.3 (6.5)	116.2 (6.6)	98.5 (7.0)

Values are mean (SE). The a, b, and c represent the significant difference compared to 23, 26, and 34 °C, respectively (*P* < 0.05). The RF and VL are the abbreviations of the rectus femoris and the vastus lateralis, respectively

*%MVC* percentages of maximum voluntary contraction

### Submaximal knee extension

**Fig. 3:**
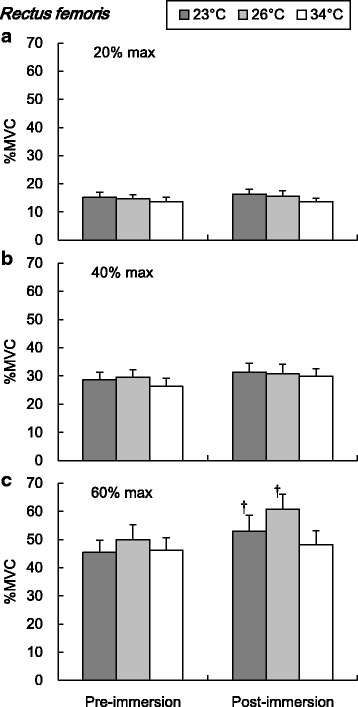
Amplitude of the electromyography in the rectus femoris during submaximal isometric knee extension. Values are mean (SE). *Dagger* represents a significant difference between pre- and post-immersion (*P* < 0.05). *%MVC* percentages of maximum voluntary contraction

**Fig. 4:**
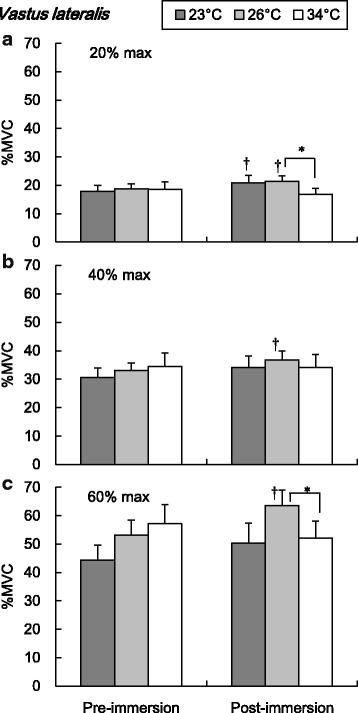
Amplitude of the electromyography in the vastus lateralis during submaximal isometric knee extension. Values are mean (SE). *Dagger* represents a significant difference between pre- and post-immersion (*P* < 0.05). *Asterisk* represents a significant difference between water temperature conditions (*P* < 0.05). *%MVC* percentages of maximum voluntary contraction

The EMG amplitude (%MVC) of RF and VL during submaximal isometric knee extension are shown in Figs. 3 and 4, respectively. The post-immersion %MVC of RF was significantly higher than pre-immersion during 60% maximal knee extension in 23 and 26 °C conditions (*P* < 0.05, Fig. 3c). In the VL, the post-immersion %MVC was significantly higher than pre-immersion in 23 and 26 °C conditions during 20% maximal exercise and in 26 °C during 40 and 60% maximal intensities (*P* < 0.05, Fig. 4a–c). The post-immersion %MVC of VL was significantly higher in 26 °C than in 34 °C during 20 and 60% maximal knee extensions (*P* < 0.05, Fig. 4a, c).

**Fig. 5:**
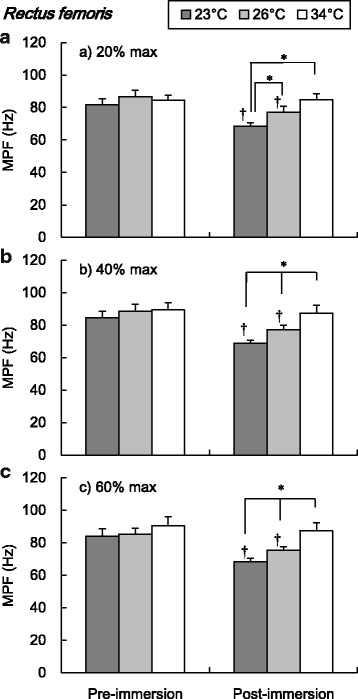
Mean power frequency of electromyography in the rectus femoris during submaximal isometric knee extension. Values are mean (SE). *Dagger* represents a significant difference between pre- and post-immersion (*P* < 0.05). *Asterisk* represents a significant difference between water temperature conditions (*P* < 0.05). *MPF* mean power frequency

**Fig. 6:**
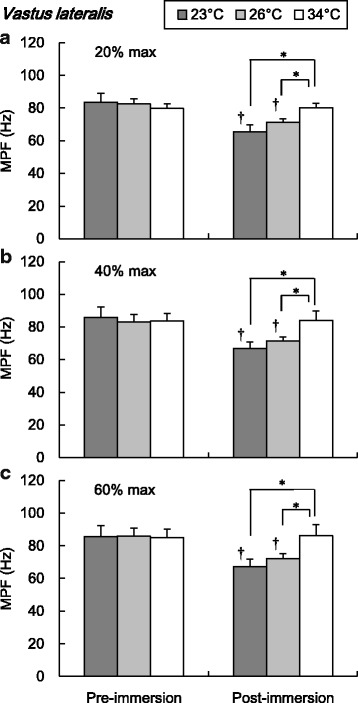
Mean power frequency of electromyography in the vastus lateralis during submaximal isometric knee extension. Values are mean (SE). *Dagger* represents a significant difference between pre- and post-immersion (*P* < 0.05). *Asterisk* represents a significant difference between water temperature conditions (*P* < 0.05). *MPF* mean power frequency

The EMG mean power frequencies (MPF) of RF and VL during submaximal isometric knee extension are shown in Figs. 5 and 6, respectively. The MPF of RF and VL in 23 and 26 °C conditions were significantly lower in post-immersion than pre-immersion trials in 20, 40, and 60% maximal intensities (*P* < 0.05, Figs. 5 and 6). The post-immersion MPF of RF during 20% maximal intensity was significantly lower in 23 °C than in 26 and 34 °C conditions (*P* < 0.05, Fig. 5a) and significantly different between three water temperature conditions during 40 and 60% maximal intensities (*P* < 0.05, Fig. 5b, c). The post-immersion MPF of VL during three submaximal trials were significantly lower in 23 and 26 °C than in 34 °C conditions (*P* < 0.05, Fig. 6a–c).

## Discussion

In this study, the effect of cold water immersion on the neuromuscular function during knee extension at maximal and submaximal intensities was assessed using the EMG technique. The maximal force after immersion was significantly lower in the 23 °C condition compared to the thermoneutral 34 °C condition, whereas no difference was observed in the amplitude of EMG. During submaximal exercises, the amplitude of EMG was higher, and the power frequency was lower after cold water immersion compared to the thermoneutral condition.

Bergh and Ekblom [25] tested the influence of muscle temperature on maximal muscle strength of knee extension exercise like our study. They reported a small decrease of MVC during isometric knee extension within the range of muscle temperature 30 to 39 °C. In the present study, the reduction of the MVC was only observed after 23 °C cold water immersion, whereas no difference was observed between in 26 °C and thermoneutral conditions (Table 1). Based on previous researches and review articles, a muscle temperature of 27 °C is assumed to be a critical temperature for initiating reduction in the maximal isometric voluntary contraction [14, 15, 17, 26, 27]. Thus, probably the muscle temperature after 60 min immersion in 23 °C water might be below the critical muscle temperature for performing MVC. Additionally, since shivering was observed during immersion in 23 °C water, the post-immersion data in 23 °C condition might include the effect of muscle fatigue due to the shivering.

During submaximal isometric knee extension, significantly lower EMG frequency of the RF and VL was observed in colder water temperature conditions (Figs. 5 and 6). The lower shift of EMG frequency with lower muscle temperature has been reported a lot [18–22]. The lower shift of EMG frequency in the cold has been regarded as a result of the decrease in the nerve and muscle fibers conduction velocity [20, 28, 29]. Regarding the amplitude of EMG, there has been more variable observation in references which reported increased [19, 23] or decreased [18, 22, 24] amplitudes in cold environment. The discrepancy could be explained by different experimental protocols including exercise type, cooling procedure, and measured muscle groups. In this study, significant increase of the EMG amplitude was observed in both RF and VL in cold water conditions (Figs. 3 and 4). In detail, the effect of cold on the EMG amplitude was more clearly observed in the VL compared to the RF. The increase of the EMG amplitude might indicate the more muscle fiber recruitment for maintaining the given work load [30], since the function of each muscle fiber is suppressed due to the cold. In this study, the lower MPF in cold would reflect the suppression of the muscle fibers conduction velocity [20, 29]. To compensate for the impairment of each muscle fibers function, more muscle fibers might be recruited to maintain the working load. This might result in the greater amplitude of EMG after the cold immersion, especially more in the VL rather than in the RF. The greater percentage of the fast twitch fiber in VL rather than in the RF [31] might explain the pronounced observation in the VL, since the role of fast twitch fiber is relatively more important in cold than in the thermoneutral condition, as explained below.

It has been reported that slow twitch fibers showed a greater reduction in the shortening velocity than fast twitch fiber in vitro [32], and faster muscle fibers are recruited at relatively lower velocity in cold water to maintain swimming speed [33, 34]. Since the slow twitch fibers have greater cold sensitivity and lower power output in cold, less cold sensitive and more powerful faster type fibers are recruited. Therefore, a greater number of fast twitch fibers need to be recruited in cold to generate the muscle powers for maintaining the work load. The recruitment of the fast twitch fiber in hypothermic skeletal muscle has been indirectly investigated in a study on muscle metabolism in cold [35]. The skeletal muscle oxygen uptake, which was assessed using near infrared spectroscopy, during isometric hand grip was gradually decreased as a function of the muscle temperature reduction [35]. This result would indicate a greater anaerobic contribution in the hypothermic skeletal muscle for maintaining the same work load as in the normothermic condition. Another report also supported the greater contribution of anaerobic metabolism with an evidence of greater minute ventilation per unit oxygen uptake (*V*_E_/*V*O_2_) in cold water during incremental cycling exercise [36], since the *V*_E_/*V*O_2_ is known to increase above the anaerobic threshold [37].

Based on the observed neuromuscular function in the hypothermic skeletal muscle described above, a potential hypothesis of cold adaptation after physical activity in cold is discussed in this paragraph. It would be some kind of cross adaptation between exercise and cold environment. Concerning the greater recruitment of faster muscle fibers and anaerobic metabolism with hypothermic skeletal muscle [33–36], the repeated physical activity with reduction of muscle temperature might improve the anaerobic exercise tolerance and function of the faster type muscle fibers. There is an interesting research on the muscle fibers characteristic of Korean diving women who have routinely engaged in physical work in cold water [10]. Divers had a greater proportion of type IIx and fewer type IIa fibers compared to control group, whereas no group difference was in the type I fibers [10]. This result suggested that repeated physical activity in cold water might induce the shift of type II muscle fibers to the faster subgroup. However, since the diving work in cold water includes comprehensive effect of hypoxia induced by the intermittent breath holdings, further controlled researches are required to clarify the cross adaptation between exercise and cold.

## Conclusions

This study investigated the effect of cold water immersion on the exercise performance and neuromuscular function. The maximal force was significantly lowered after cold water immersion. The lower shift of EMG frequency and greater EMG amplitude was observed in the femoral skeletal muscle during submaximal knee extension exercise after immersion in cold water.

## Abbreviations

%MVC: Percentages of maximum voluntary contraction
EMG: Electromyography
MPF: Mean power frequency
MVC: Maximal voluntary contraction
RF: Rectus femoris
RMS: Root mean square
VL: Vastus lateralis
